# Supplementary material for: The Treatment of Metabolic Acidosis: An Interactive Case-Based Learning Activity
Source: MedEdPORTAL. 2019 Sep 27;15:10835. doi: 10.15766/mep_2374-8265.10835 (PMC6897540; doi:10.15766/mep_2374-8265.10835)
Supplement: Supplementary file 1 — A. Approach to Acid-Base Disorders.mp4 B. Tale of Two Acidoses.mp4 C. IRAT Quiz.docx D. IRAT Quiz KEY.docx E. In-Class Cases.docx F. In-Class Cases Instructor Guide.docx [file mep-15-10835-s001.zip › _Educational Summary Report_10835.pdf]

MedEdPORTAL® The AAMC Journal of Teaching and Learning Resources

Original Publication Open Access

# The Treatment of Metabolic Acidosis: An Interactive Case-Based Learning Activity

Michael Berkoben, MD, John K. Roberts, MD, MS, MEd*

*Corresponding author: john.roberts@duke.edu

## Abstract

**Introduction:** Metabolic acidosis is a dangerous and potentially life-threatening condition encountered in the inpatient and emergency department setting. Metabolic acidoses due to renal failure, bicarbonate losses, or lactic acidosis are common conditions, and the appropriate medical management of each is relevant to any inpatient medical provider. Therefore, we created a learning activity that utilizes blackboard-style videos followed by an interactive case-based learning session to help the medical student recognize, diagnose, and manage common causes of metabolic acidosis. **Methods:** We organized this learning activity by assigning digital videos, followed by application in an interactive team-based format. We created electronic blackboard-style videos and a quiz to assess medical knowledge related to concepts discussed in the videos. Next, we created case resources that facilitate an interactive case-based teaching session so the learners could apply their knowledge and simulate the management of metabolic acidosis. **Results:** We implemented this activity for 34 medical students. All students viewed the videos prior to the in-class session. In a pre/post assessment of medical knowledge, we observed a significant improvement in quiz scores. Next, we successfully facilitated the case-based active learning session, allowing the assessment of higher-order cognitive skills related to management of patients with metabolic acidosis. Our medical students felt highly satisfied and competent at the completion of our course. **Discussion:** Our medical students rated this as an excellent learning activity. Others may find this activity useful within the context of any course or rotation related to patients with metabolic acidosis.

**Keywords**
Metabolic Acidosis, Physiology, Nephrology, Lactic Acidosis, Renal Failure, Critical Care Medicine, Emergency Medicine, Nephrology, Flipped Classroom

## Educational Objectives

By the end of this activity, learners will be able to:

1. Identify metabolic acidosis.
2. Discriminate between anion gap and nonanion gap metabolic acidosis.
3. Identify the rate of acid production from the diet.
4. Identify the composition of isotonic bicarbonate solution.
5. Identify how acid-base balance affects serum potassium concentrations.
6. Define simple and complex acid-base disorders given a patient's laboratory values.
7. Write orders for the rational administration of bicarbonate in severe metabolic acidosis.
8. Write orders for a patient with metabolic acidosis due to lactic acidosis.

**Citation:**
Berkoben M, Roberts JK. The treatment of metabolic acidosis: an interactive case-based learning activity. *MedEdPORTAL.* 2019;15:10835. https://doi.org/10.15766/mep_2374-8265.10835

## Introduction

This educational activity was produced to teach medical students the fundamental knowledge and skills necessary to diagnose and treat metabolic acidosis. Other learning resources related to metabolic acidosis exist; however, most of these resources are geared solely toward the identification and management of toxin ingestions.[1-5] Although these are clinically important medical emergencies, metabolic acidosis due to toxin ingestion remains a relatively rare clinical encounter. Metabolic acidoses due to renal failure, bicarbonate losses, or lactic acidosis are much more common conditions, and the appropriate medical management of each is relevant to any inpatient medical provider. Therefore, there is a gap between the availability of high-quality learning resources and what is most commonly experienced clinically when it comes to metabolic acidosis. In response, we created a learning activity that better represents the management of

Copyright © 2019 Berkoben and Roberts. This is an open-access publication distributed under the terms of the Creative Commons Attribution license.

metabolic acidosis due to a wide variety of common causes, with special attention to the administration of sodium bicarbonate.

This activity is designed such that didactic content is first delivered through new digital blackboard-style videos, then learners apply their knowledge in an interactive case-based learning activity. The ubiquity of access to the internet and other technologies makes videos convenient for the current generation of learners. Digital video can be watched repeatedly and at whatever pace the learner chooses. The video as a learning resource allows classroom time to be better spent on active learning strategies as opposed to live large-group lectures. Active learning in the place of live lectures has been associated with improved course performance in undergraduate science, technology, engineering, and math courses and improved attention during live tutorials.[6,7]

Other learning resources are available for teaching medical students about acid-base physiology and associated disorders. Some resources include representative acid-base cases that can be used in a team-based learning format or facilitated case-based lecture.[8-10] These case-based resources could easily be implemented within an existing course or clinical clerkship; however, all but one of these published resources lack an assessment of student knowledge. Some of the newer, more engaging resources are simulations of acute toxin ingestions, which require a high-fidelity patient simulator, medical equipment, and multiple faculty members.[1-5] Many of these simulation-based resources are focused on higher-level cognitive and procedural competencies surrounding the acute management of a patient in the emergency department. These simulations mostly target emergency department and pediatrics residents, as they are training to be team leaders in the acute care setting. Medical student education, however, needs to accommodate a large group of learners, and many schools may not have the funds or expertise to run simulations on such a large scale. In light of the available published resources, our activity provides a complete and robust learning experience that includes digital videos, an assessment of learning, and interactive case-based application. Our activity encompasses the entire spectrum of metabolic acidosis (common causes as opposed to focusing only on rare ingestions), includes a readiness assessment, and is designed to prepare a fourth-year medical student for direct patient care after graduation (by writing patient care orders).

## Methods

### Overview

This learning activity comprised a complete work cycle surrounding basic concepts regarding the recognition and management of metabolic acidosis. This activity was designed to work as a stand-alone activity for the learner or as a portion of a larger course. In addition, the activity comprised work done prior to the classroom session, which was then followed by activities occurring during a 2-hour in-class session. We implemented this activity with a classroom size of 30 to 35 students.

### Development of Learning Activities

All of the components presented in this learning activity were implemented and vetted with medical students prior to incorporation in the course. The “Approach to Acid-Base Disorders” video (Appendix A) was created by us and implemented in our school’s first-year medical school curriculum in a medical physiology course. This video was based directly on large-group lecture material that had been delivered for many years with positive feedback. The content in “Approach to Acid-Base Disorders” likely was not new to our fourth-year students; however, we included it in this activity as a refresher of basic science concepts to consolidate prior learning. Next, we created the “Tale of Two Acidoses” video (Appendix B) after extensive personal use and positive feedback from medical students and residents. We wrote a script and personally created the images seen in the video. The content in “Tale of Two Acidoses” was based on two actual patient encounters. One author (Dr. Berkoben) was the treating physician in both cases. In addition, the content was based on a large-group slide-based presentation that he has been giving to medical students and internal medicine residents for many years. Based on evaluations and feedback, it was clear that these cases were useful and provided important concepts surrounding the management of patients with metabolic acidosis. In an effort to reduce large-group presentations and use class time for active case-based learning, we decided to convert his large-group presentation into a digital blackboard video. From a technical standpoint, both videos were created using the following software and hardware: a Bamboo tablet (Wacom Technology Corporation, Portland, Oregon), a Snowball microphone (Blue Microphones, Westlake Village, California), Screencast-O-Matic (Screencast-O-Matic, Seattle, Washington), and SmoothDraw, version 4.0.1. Dr. Berkoben created the video script and content, and Dr. Roberts created the drawings and handled technical aspects of the recording process. After creating both videos, each was implemented in medical physiology courses and our nephrology fellow core curriculum. In both instances, we received feedback that the videos were clear, concise, and excellent resources that reflected real clinical scenarios. After implementation, we have not had to make changes to the videos prior to using them in the Fluids and Electrolytes course. The reviews and evaluations thus

Copyright © 2019 Berkoben and Roberts. This is an open-access publication distributed under the terms of the Creative Commons Attribution license.

far have been very positive, so we do not plan any revisions to these videos.

The individual readiness assessment test (IRAT; Appendix C) was created specifically for implementation in this course. Prior to the study described in this report, we created the IRAT through an iterative process. First, we reviewed the learning objectives for our activity. Dr. Berkoben then created the questions to match the learning objectives that were solely based on medical knowledge (as higher-level learning objectives are tested in the interactive sessions). The set of questions was reviewed by Dr. Roberts and edited for clarity and appropriateness. We revised the IRAT to make answer choices homogenous in content and to include plausible distractors. At the conclusion of the session, no students raised objections regarding the clarity or appropriateness of these questions.

Regarding the cases used in the interactive case-based sessions, all cases were based on actual patient encounters. Cases 1, 2, and 3 were based on actual patients Dr. Roberts cared for while a nephrology fellow at Duke University Medical Center. Case 4 was an actual patient cared for by one of Dr. Roberts' former teachers. These cases were chosen because they represented common problem presentations on the general medicine wards and intensive care units. We have been utilizing these cases for about 5 years in the Fluids and Electrolytes course. Our students have cited these cases as being clinically accurate and effective at forcing them to apply their knowledge in a collaborative, team-based setting. Throughout implementation over 5 years, no students have raised concerns regarding the clarity or appropriateness of these cases.

### Implementation of Learning Activities

*Blackboard videos:* We implemented this learning activity on a weekly cycle as part of a larger course. For example, our in-class learning session occurred on a Wednesday afternoon. At the conclusion of the prior week's events, the students were required to watch two tutorial videos: "Approach to Acid-Base Disorders" (Appendix A) and "Tale of Two Acidoses" (Appendix B). These videos could have been viewed on any computer or electronic device with internet access and a media player. The videos were hosted on a Vimeo account, and the video links/documents were arranged in sequence on a Google Classroom webpage. The students were required to watch the videos between the conclusion of the last session and the next scheduled session, so they had 6 days to complete this assignment.

*Individual readiness assessment:* At 5:00 p.m. 2 days prior to the large-group learning activity, the IRAT (Appendix C) was activated (available as a Google Form on Google Classroom) and available for completion. Considering that the large-group session started at 5:00 p.m., the students had 48 hours to complete the assessment before it was deactivated at the start of the in-class activity. After completion of the quiz, students immediately reviewed their performance. The content of the IRAT mapped directly to the first five learning objectives for this activity. The IRAT key (Appendix D) was available to the faculty member and could be given to the students after the quiz was completed.

*Case-based activity:* The in-class learning activity took place in a classroom large enough for 40 students. The room was equipped with a projector and a projector screen (which could be moved up or down), and two entire walls of the room were whiteboard space equipped with dry-erase markers and erasers. The classroom included large rolling tables, which could easily be configured to accommodate multiple smaller groups of students. The students in attendance were divided into small teams: typically, three to four students comprising six groups of four students and two groups of three students.

At the start of the activity, each team was given a chance to review the IRAT. Each question was shown to the class (projected on the screen), and we then asked the students to identify the correct answers. Some questions were quite easy, and all teams identified the correct answer. Some questions generated multiple answers, and this is where we asked students to justify their responses. Based on student questions and knowledge gaps, we took this opportunity to explain the concepts underlying the question, using the whiteboard as needed to fully cover the learning objectives. This process allowed for spaced repetition of key concepts, and it permitted us to personalize the teaching toward only the difficult or confusing concepts. This process took about 15-20 minutes.

Next, we began the interactive case-based teaching session. Each team was given the same series of four cases (Appendix E), and each student had a paper copy. Each case provided a brief clinical history, vital signs/physical examination, and laboratory data. Each team was required to collaboratively solve the acid-base disorder, followed by writing the actual management orders (only those related to the acid-base disorder) as if the student team were the primary admitting physician for the patient. The students also needed to identify the most likely mechanism for the disturbance. The potential mechanisms for a metabolic acidosis were covered in Appendix B, which students should have viewed prior to class. The students needed 45 minutes to complete all four cases in teams.

Copyright © 2019 Berkoben and Roberts. This is an open-access publication distributed under the terms of the Creative Commons Attribution license.

Next, we reviewed each case as a large group. The facilitator/instructor called on teams to name the acid-base disorder, describe their orders, and propose a mechanism for the disorder. We asked teams to justify their orders especially if the orders were not specific (e.g., “give bicarbonate”), and then we asked them to elaborate and give specifics on the dose, volume, and route of administration of the medication or fluid. The instructor’s guide to the cases should be reviewed prior to facilitating this session (Appendix F). The cases represented the four major categories of metabolic acidosis by mechanism: chronic renal failure, acute lactic acidosis, bicarbonate losses (due to diarrhea), and ingestion (due to salicylate toxicity). This activity generated discussion surrounding the management of patients with metabolic acidosis. The students mentioned things they had seen or heard on clinical rotations. This provided an amazing opportunity for us to clarify concepts, dispel myths, and reinforce concepts. The interactive case-based learning session allowed the students to meet educational objectives 6 through 8 related to higher-order cognitive skills such as defining simple and complex acid-base disorders, writing orders for the rational administration of bicarbonate, and writing orders for a patient with lactic acidosis.

Sample Schedule for the In-Class Session

- 5:00-5:20 p.m.: Review readiness assessment questions/answers as large group.
- 5:20-6:05 p.m.: Students work in teams on the four cases (Appendix E).
- 6:05-7:00 p.m.: Facilitated discussion of the cases and team responses/orders.

## Results

We implemented this learning activity as part of our Fluids and Electrolytes elective for fourth-year students in the winter of 2018. Thirty-four fourth-year medical students enrolled in the course and completed this activity. The facilitator was a faculty member at the Duke University School of Medicine and a nephrologist at the Duke University Medical Center. The instructor had experience with facilitating active learning sessions and team-based activities.

We measured the effectiveness of our digital blackboard videos (Appendices A and B) on the medical knowledge learning objectives. We administered the 11-question IRAT to our fourth-year medical students ($N = 34$) prior to the session devoted to acid-base homeostasis and metabolic acidosis. As usual, students were assigned to watch the videos (Appendices A and B) and then attend the in-class clinical problem-solving session in teams. Two days following the in-class clinical problem-solving session, students were again invited to take the IRAT. For each quiz, the mean and standard deviation were calculated for continuous data. An unpaired $t$ test was used to compare the means of the two groups. A two-tailed $p$ value of less than .05 was defined as a significant difference in the means. Analyses were performed in STATA, version 14.0.

When the resource was implemented in 2018, 100% of our students viewed the two tutorial videos prior to the classroom session. When we used the videos (Appendices A and B) in conjunction with a case-based active learning session, medical knowledge improved significantly as assessed by the pre/post quiz. Prior to the video content, participating students ($n = 18$) had a mean quiz score of 6.7 ($SD = 1.3$) out of 11 total points (61% correct). After viewing the video and attending our session, the participating students ($n = 13$) had a mean quiz score of 8.8 ($SD = 1.3$) out of 11 (80% correct). The difference in mean quiz scores was significant ($p < .001$).

During the in-class active learning session, we evaluated the students’ abilities to meet the higher-order learning objectives: solving acid-base disorders, writing orders for each case, and identifying the mechanism of acidosis. All participating students were able to complete the activity and correctly identify the acid-base disorder in each case. For the part of the activity where students wrote orders, we observed some variability in the responses, providing an excellent opportunity for retrieval practice and correcting mistakes. For example, common teaching points include ordering blood tests to work up an anion gap metabolic acidosis (e.g., plasma lactate concentration and plasma beta-hydroxybutyrate concentration). In addition, some teams wrote very brief or limited orders (e.g., “give bicarbonate”), and we used this opportunity to elaborate on the order and provide specifics on the dose, volume, and route of administration of the medication. Therefore, we have found that real-time assessment of the application (based on student answers/responses) is an effective way to assess the utility of this learning activity.

Next, we examined the durability of this learning activity through performance on acidosis-themed questions on the final examination. The Fluids and Electrolytes final examination was administered 4 weeks after the metabolic acidosis interactive case-based session. The examination consisted of 37 question items with a mix of multiple-choice questions, matching, and narrative response. The final examination questions were all case based and much more complex compared to the IRAT. Seven examination items were directly based on learning objectives

Copyright © 2019 Berkoben and Roberts. This is an open-access publication distributed under the terms of the Creative Commons Attribution license.

covered by this learning activity. In the recent cohort of fourth-year medical students ($N = 34$), the mean percentage correct on the final examination was 91.0 ($SD = 5.6$), whereas the mean percentage correct on acidosis-themed questions was 97.0 ($SD = 5.5$). The difference in these mean scores was statistically significant ($p < .0001$). This shows that our students performed better on the acidosis-themed questions compared to the other items on the final examination. Therefore, our learning activity resulted in sustained and substantial learning that was reflected on the final examination given 1 month after the learning activity. In prior iterations of our course, final examination scores were very similar overall: mean scores of 92.3 ($SD = 5.0$) in 2017 and 91.1 ($SD = 5.3$) in 2016. Although the course learning objectives and final examination did not change compared to prior years, the exact configuration of the metabolic acidosis session (as presented) did change as we included more cases in the interactive session and offloaded more didactic content to digital blackboard videos.

In our comprehensive course evaluation, we evaluated the specific sessions, including this one. Of the nine respondents, eight students (89%) felt that no changes were needed for this acid-base learning session. One respondent (11%) felt that changes were needed. In a free-text portion of the evaluation of this session, students were asked if anything else should be added to it. Two students thought nothing needed to be added. One student responded, "More clinical cases and walkthroughs of those cases/practical advice." In addition, to better understand the acceptability and feasibility of our metabolic acidosis learning activity, we asked our student cohort to participate in a targeted survey. Students were given open-ended prompts to specifically comment on perceived utility, acceptability, and overall satisfaction in our combination of digital blackboard videos followed by the IRAT and interactive case-based learning session about metabolic acidosis. Responses included the following:

- Student 1: "The videos were excellent. They taught basic principles of physiology and pathophysiology in a straightforward and easy to conceptualize way. They reinforced things I learned previously and solidified my understanding. Having the practical cases after having reviewed the videos was also excellent. It allowed me to put into practice, in a very meaningful way, the concepts that I had learned in the videos. Was an invaluable experience that I think will be highly useful and beneficial as a practicing clinician."
- Student 2: "I felt that the presession videos were extremely helpful in laying a foundation that allowed me to work through and actually learn from the cases. The videos provided the basic knowledge (physiology etc.) and the case taught me how to apply my basic science knowledge to the clinic. I thought the videos were of good length and the right amount of depth and I liked having them prior to working through the cases. They were not too advanced and not too easy either. I really enjoyed having the cases, as I believe you should practice how you expect to play. The quizzes were good at motivating me [to] pay attention during the videos but I felt the quiz questions may have been too simple to actually promote retained knowledge. That said the final was more difficult and better at pulling out what I understood and retained and also helped me integrate knowledge from throughout the course."
- Student 3: "From my perspective, the integrated videos and quizzes was a superb teaching method for retention and knowledge application. The short, to the point quizzes (not trying to trick you) allowed me to apply the take home points of the videos. The videos were especially valuable during fourth year, as this allowed me to efficiently study when traveling for interviews and still be involved with the class when I had to miss a session. As expected, most of the learning occurred in class, where we applied what we had learned to cases. I think the method of videos, quizzes, and cases in class will help me retain the information necessary to better care for my patients intern year."

Overall, we have demonstrated that our bundled learning activity (combination of digital blackboard videos, followed by an IRAT, then interactive case-based sessions) was effective in teaching knowledge and higher-order cognitive skills related to treatment of metabolic acidosis. We have shown that our learning activity impacted both short-term performance and performance on the final examination administered 4 weeks later. Based on evaluation data and a targeted survey, students who participated in this activity found it flexible, useful, and highly applicable to clinical care. The students thought the videos were effective and appropriately constructed, and they greatly enjoyed applying their knowledge in class with high-fidelity cases.

## Discussion

This learning activity was successful when we implemented it as part of a larger course for fourth-year medical students. The series of digital videos, readiness assessment, and cases allows the learner to review material in a personalized format, engage in retrieval practice, then apply knowledge in an active learning session with activities very similar to actual patient care. To initially implement this activity, we decided to offload content

Copyright © 2019 Berkoben and Roberts. This is an open-access publication distributed under the terms of the Creative Commons Attribution license.

that traditionally had been delivered as a large-group lecture into blackboard tutorial videos. The video tutorial is a popular format among the current generation of learners. The video can be viewed on a smartphone or tablet, and the viewer can learn from the video at a customized pace. Studies analyzing the best practices for instructor-led videos have been performed. The electronic blackboard tutorial has been associated with high levels of student engagement.[11] Videos such as this have proved to be very popular among our medical students, residents, and fellows.

Overall, our students and instructors were pleased with the sequence of learning activities, and both found the cases to be realistic. We believe that this is an excellent educational resource for medical students or any learner who encounters patients with metabolic acidosis. This activity addresses what we perceive to be a gap in acid-base learning resources: Relatively less attention is given to managing metabolic acidosis due to bicarbonate losses, renal failure, or lactic acidosis, all of which are much more common than toxic ingestions.

In this activity, we have a sequence where the learner can obtain the proper knowledge and then apply it by solving cases and placing orders. This learning cycle truly achieves its purpose through the interactive case-based teaching session. Here, the instructor is able to directly assess each team's ability to meet the specified learning objectives. Because each group of students may have difficulty with different concepts, the proposed activity allows the appropriate flexibility to respond to students' unique needs and knowledge gaps. As discussion is generated for each case, the instructor can immediately assess whether or not students are meeting the prespecified higher-order learning objectives of this activity.

This learning activity in its current form represents the final version of an activity we have been implementing for 5 years. Over that time, we have incorporated student and instructor feedback to finally arrive at the current version of the activity. The key changes revolve around the use of time during the in-class portion of the activity. Initially, we would break the class up into small teams (three to four students each) and work through cases as a large group (cases were presented from the projector), with an assigned team solving its case in real time and presenting to the large group. In response to feedback, we found that students preferred to work collaboratively on all of the cases for a time, followed by discussion/solution time with the instructor. Therefore, this version reflects this format for the interactive case-based teaching session. We believe that this format allows better student engagement across the classroom and offers more opportunities for retrieval practice for each student.

There are several limitations worth noting. First, this activity requires that the students have sustained access to a computer or other smart device to view the videos during the week before class. Second, the activity is most successful with a room that contains modular tables that can be broken apart for the small teams (three to four students each). Therefore, space and equipment could limit the ability to facilitate this session. In addition, because our course is designed to run concurrently with clinical rotations, we implement this session in the late afternoon (5:00-7:00 p.m.). Thus, to ensure student enrollment and attendance, the in-class activity needs to be conducted at a time that minimizes conflicts with clinical rotations. For evaluation and feedback, the response rate was 26%; therefore, there could have been a response bias such that the responses did not reflect the entire cohort of students. Finally, the results seen at our institution may not be generalizable to other institutions. For example, if treatment of metabolic acidosis/use of sodium bicarbonate is taught elsewhere in the curriculum during the fourth year of medical school, this learning activity may be redundant. However, because of the clinical significance and incidence of metabolic acidosis in hospitalized patients, spaced repetition/interleaving of this content is recommended. Additionally, we received constructive feedback from a student who recommended "more clinical cases and walkthroughs of those cases." We have found that four cases and discussion are the optimal amount of content for the timing of our session. A fifth case could certainly be added, although only at the sacrifice of reviewing the quiz and any additional ad hoc tutorial in response to student scores. We believe that this review of the quiz is necessary to identify and correct knowledge gaps prior to moving to the active learning phase of the activity. If more cases need to be added, then the session length would need to be extended beyond 2 hours.

We will continue to use this learning activity in our fourth-year Fluids and Electrolytes course. We believe that the digital blackboard videos serve as an excellent tutorial for solving acid-base disorders and identifying/treating metabolic acidosis. Based on our experience, we have found that the videos, in conjunction with an interactive case-based teaching session, lead to improved medical knowledge and high levels of student satisfaction. Our students self-report feeling more prepared for clinical care. This format (using digital blackboard videos followed by interactive case-based teaching sessions) has

Copyright © 2019 Berkoben and Roberts. This is an open-access publication distributed under the terms of the Creative Commons Attribution license.

become the standard format for the other sessions in our Fluids and Electrolytes course.

To better understand the impact of our intervention, a randomized study comparing digital videos/case-based sessions to other learning formats (live lectures or self-directed modules) would need to be performed. In addition, we would like to provide continued access to our tutorial videos throughout the intern year (postgraduate year 1) and measure the usefulness and effectiveness of these resources at the point of care. In future studies, we would like to study the impact of this learning activity on self-reported outcomes and patient outcomes associated with our students during their intern year of residency.

## Appendices

A. Approach to Acid-Base Disorders.mp4
B. Tale of Two Acidoses.mp4
C. IRAT Quiz.docx
D. IRAT Quiz KEY.docx
E. In-Class Cases.docx
F. In-Class Cases Instructor Guide.docx

*All appendices are peer reviewed as integral parts of the Original Publication.*

**Michael Berkoben, MD:** Associate Professor, Department of Medicine, Division of Nephrology, Duke University Medical Center

**John K. Roberts, MD, MS, MEd:** Assistant Professor, Department of Medicine, Division of Nephrology, Duke University Medical Center

**Disclosures**
None to report.

**Funding/Support**
None to report.

**Ethical Approval**
Reported as not applicable.

## References

1. Lammers RL, Sheakley ML, Hendren S. A simulated case of acute salicylate toxicity from an intentional overdose. *MedEdPORTAL*. 2018;14:10678. https://doi.org/10.15766/mep_2374-8265.10678
2. Wittler M, O'Brien MC, Masneri DA. Toxic alcohol ingestion/methanol ingestion. *MedEdPORTAL*. 2018;14:10740. https://doi.org/10.15766/mep_2374-8265.10740
3. Wittler M, Masneri DA, Hannum J. Chronic salicylate toxicity simulation. *MedEdPORTAL*. 2018;14:10741. https://doi.org/10.15766/mep_2374-8265.10741
4. Mitzman J, Wittler M. Toxic ingestion: anion gap metabolic acidosis caused by acute salicylate ingestion. *MedEdPORTAL*. 2014;10:9893. https://doi.org/10.15766/mep_2374-8265.9893
5. Burns R, Reid J, Mazor S. Pediatric Toxidrome Simulation Curriculum: salicylate toxidrome. *MedEdPORTAL*. 2014;10:9913. https://doi.org/10.15766/mep_2374-8265.9913
6. Freeman S, Eddy SL, McDonough M, et al. Active learning increases student performance in science, engineering, and mathematics. *Proc Natl Acad Sci U S A*. 2014;111(23):8410-8415. https://doi.org/10.1073/pnas.1319030111
7. Bunce DM, Flens EA, Neiles KY. How long can students pay attention in class? A study of student attention decline using clickers. *J Chem Educ.* 2010;87(12):1438-1443. https://doi.org/10.1021/ed100409p
8. LeClair RJ, Binks AP. Integrating acid-base and metabolic lab panels across systems in an M1 classroom activity. *MedEdPORTAL*. 2019;15:10793. https://doi.org/10.15766/mep_2374-8265.10793
9. Bishop S, Kushinka J, Call S. Internal medicine clerkship team-based learning series: acid-base module. *MedEdPORTAL*. 2012;8:9177. https://doi.org/10.15766/mep_2374-8265.9177
10. Raghavan R. Understanding electrolytes: 15 interactive teaching cases. *MedEdPORTAL*. 2013;9:9537. https://doi.org/10.15766/mep_2374-8265.9537
11. Guo PJ, Kim J, Rubin R. How video production affects student engagement: an empirical study of MOOC videos. In: *L@S 2014: Proceedings of the First ACM Conference on Learning @ Scale*. Atlanta, GA: Association for Computing Machinery; 2014:41-50. https://doi.org/10.1145/2556325.2566239

**Received:** December 18, 2018
**Accepted:** May 21, 2019
**Published:** September 27, 2019

Copyright © 2019 Berkoben and Roberts. This is an open-access publication distributed under the terms of the Creative Commons Attribution license.
