# Supplementary material for: The Treatment of Metabolic Acidosis: An Interactive Case-Based Learning Activity
Source: MedEdPORTAL. 2019 Sep 27;15:10835. doi: 10.15766/mep_2374-8265.10835 (PMC6897540; doi:10.15766/mep_2374-8265.10835)
Supplement: Supplementary file 1 — A. Approach to Acid-Base Disorders.mp4 B. Tale of Two Acidoses.mp4 C. IRAT Quiz.docx D. IRAT Quiz KEY.docx E. In-Class Cases.docx F. In-Class Cases Instructor Guide.docx [file mep-15-10835-s001.zip › C. IRAT Quiz.docx]

**The Treatment of Metabolic Acidosis: An Interactive Case-based Learning Activity.**

**Individual Readiness Assessment**

1. Metabolic acidosis is characterized by
2. Low arterial pH
3. Reduced plasma bicarbonate concentration
4. Compensatory hypoventilation
5. a and b
6. Which of the following lead(s) to an increase in the anion gap?
7. Diabetic ketoacidosis
8. Diarrhea
9. Renal failure
10. a and c
11. The distribution space of bicarbonate
12. Remains constant as metabolic acidosis becomes more severe
13. Decreases as metabolic acidosis becomes more severe
14. Increases as metabolic acidosis becomes more severe
15. The daily dietary hydrogen ion load is
16. Negligible
17. Roughly 50-100 mEq per day
18. Several hundred mEq per day
19. A mineral acid load (as with decreased acid excretion in chronic kidney disease) will lead to
20. No change in plasma potassium
21. A decrease in plasma potassium
22. An increase in plasma potassium
23. In patients with severe acidemia, the initial goal is to raise the arterial pH to
24. 7.10
25. 7.20
26. 7.30
27. 7.40
28. An isotonic solution of sodium bicarbonate contains
29. 75 mEq sodium and 75 mEq bicarbonate
30. 150 mEq sodium and 150 mEq bicarbonate
31. 300 mEq sodium and 300 mEq bicarbonate
32. Which disorder has the potential to lead to the more rapid development of severe acidemia?
33. Renal failure
34. Lactic acidosis
35. The Henderson equation allows one to calculate the
36. Bicarbonate deficit
37. Rate of acid production
38. Free proton concentration in the plasma
39. Expected degree of compensatory hyperventilation in metabolic acidosis
40. The key aspect of management of patients with lactic acidosis is to
41. Administer intravenous sodium bicarbonate at a very rapid rate
42. Restore tissue oxygenation
43. Enhance anaerobic glycolysis
44. Remove lactate by hemodialysis
45. b and c
46. A young woman comes to the emergency department because of muscle paralysis. She is awake, alert, and interactive. Her past medical history is notable only for “mixed connective tissue disease.” Her plasma potassium is 1.5 mEq/L. Arterial blood gas analysis shows pH 7.02, PCO2 16, bicarbonate less than 5 mEq/L. You call the nephrology fellow. He makes the correct diagnosis of Type I renal tubular acidosis. (Type I RTA is commonly associated with autoimmune disorders). Which would you correct first?
47. Hypokalemia
48. Acidemia
