# Supplementary material for: The Treatment of Metabolic Acidosis: An Interactive Case-Based Learning Activity
Source: MedEdPORTAL. 2019 Sep 27;15:10835. doi: 10.15766/mep_2374-8265.10835 (PMC6897540; doi:10.15766/mep_2374-8265.10835)
Supplement: Supplementary file 1 — A. Approach to Acid-Base Disorders.mp4 B. Tale of Two Acidoses.mp4 C. IRAT Quiz.docx D. IRAT Quiz KEY.docx E. In-Class Cases.docx F. In-Class Cases Instructor Guide.docx [file mep-15-10835-s001.zip › D. IRAT Quiz KEY.docx]

**The Treatment of Metabolic Acidosis: An Interactive Case-based Learning Activity.**

**Individual Readiness Assessment Key**

1. Metabolic acidosis is characterized by
2. Low arterial pH
3. Reduced plasma bicarbonate concentration
4. Compensatory hypoventilation
5. a and b

Answer: d. Metabolic acidosis leads to compensatory hyperventilation.

1. Which of the following lead(s) to an increase in the anion gap?
2. Diabetic ketoacidosis
3. Diarrhea
4. Renal failure
5. a and c

Answer: d

1. The distribution space of bicarbonate
2. Remains constant as metabolic acidosis becomes more severe
3. Decreases as metabolic acidosis becomes more severe
4. Increases as metabolic acidosis becomes more severe

Answer: c. The apparent bicarbonate distribution space is about 55 percent of lean body weight in moderate metabolic acidosis and may exceed 70 percent of lean body weight in severe metabolic acidosis.

1. The daily dietary hydrogen ion load is
2. Negligible
3. Roughly 50-100 mEq per day
4. Several hundred mEq per day

Answer: b. The dietary acid load comes mainly from the catabolism of sulfur-containing amino acids.

1. A mineral acid load (as with decreased acid excretion in chronic kidney disease) will lead to
2. No change in plasma potassium
3. A decrease in plasma potassium
4. An increase in plasma potassium

Answer: c. An extracellular acid load leads to movement of hydrogen ions from the extracellular fluid to the intracellular fluid. If electroneutrality is to be preserved, an anion (such as chloride, the major extracellular anion) must move from the extracellular fluid to the intracellular fluid or a cation must move from the intracellular fluid to the extracellular fluid. Since the cell membrane is rather impermeable to chloride, a cation must exit the cell. The major intracellular cation is potassium. Therefore, a mineral acid load should lead to hyperkalemia. Therefore, c is the best answer.

1. In patients with severe acidemia, the initial goal is to raise the arterial pH to
2. 7.10
3. 7.20
4. 7.30
5. 7.40

Answer: b

1. An isotonic solution of sodium bicarbonate contains
2. 75 mEq sodium and 75 mEq bicarbonate
3. 150 mEq sodium and 150 mEq bicarbonate
4. 300 mEq sodium and 300 mEq bicarbonate

Answer: b

1. Which disorder has the potential to lead to the more rapid development of severe acidemia?
2. Renal failure
3. Lactic acidosis

Answer: b, In the setting of renal failure, metabolic acidosis develops due to the inability to excrete the acid that is produced daily. Assuming a normal diet, this is approximately 1 mEq/kg/day. For a 70kg person, this would be 70 mEq of acid added to the patient per day. In the setting of lactic acidosis, lactic acid can be generated at 36 mEq per minute if oxygen delivery is reduced by 50%. Therefore, the generation of a lactic acidosis can be much more rapid than acidosis from renal failure.

1. The Henderson equation allows one to calculate the
2. Bicarbonate deficit
3. Rate of acid production
4. Free proton concentration in the plasma
5. Expected degree of compensatory hyperventilation in metabolic acidosis

Answer: c, The Henderson equation describes the method to calculate the free proton concentration in the plasma. It is [H^+^] = 24 (pCO_2_ / HCO_3_^-^).

1. The key aspect of management of patients with lactic acidosis is to
2. Administer intravenous sodium bicarbonate at a very rapid rate
3. Restore tissue oxygenation
4. Enhance anaerobic glycolysis
5. Remove lactate by hemodialysis
6. b and c

Answer: b. The goal of management is to restore tissue oxygenation and shut off anaerobic glycolysis.

1. A young woman comes to the emergency department because of muscle paralysis. She is awake, alert, and interactive. Her past medical history is notable only for “mixed connective tissue disease.” Her plasma potassium is 1.5 mEq/L. Arterial blood gas analysis shows pH 7.02, PCO2 16, bicarbonate less than 5 mEq/L. You call the nephrology fellow. He makes the correct diagnosis of Type I renal tubular acidosis. (Type I RTA is commonly associated with autoimmune disorders). Which would you correct first?
2. Hypokalemia
3. Acidemia

Answer: a. This patient has muscle paralysis from hypokalemia. In spite of this, she is still able to lower her PCO2. Correction of acidemia with sodium bicarbonate would further lower her plasma potassium level and could produce diaphragmatic paralysis. The combination of metabolic acidosis and respiratory acidosis could prove lethal. Hypokalemia should be corrected first.
