# Supplementary material for: The Treatment of Metabolic Acidosis: An Interactive Case-Based Learning Activity
Source: MedEdPORTAL. 2019 Sep 27;15:10835. doi: 10.15766/mep_2374-8265.10835 (PMC6897540; doi:10.15766/mep_2374-8265.10835)
Supplement: Supplementary file 1 — A. Approach to Acid-Base Disorders.mp4 B. Tale of Two Acidoses.mp4 C. IRAT Quiz.docx D. IRAT Quiz KEY.docx E. In-Class Cases.docx F. In-Class Cases Instructor Guide.docx [file mep-15-10835-s001.zip › E. In-Class Cases.docx]

**The Treatment of Metabolic Acidosis: An Interactive Case-based Learning Activity.**

**Acid-Base Cases for Small Group Teaching**

**Case 1.**

- 61 year-old woman brought to ED by EMS after being found minimally responsive at home.
- Patient has not been seen by family for a few days. She has been taking NSAIDs for severe back pain.
- Medical History: chronic kidney disease, last serum creatinine 5.4 mg/dl
- EMS was called, patient was obtunded, tachycardic, tachypneic, taken to the ED.
- **Physical Examination:**
- VS: BP 100/62 mmHg; HR 110/min; RR 28/min; Temp 37.3 C; SpO2 98% on 3L NC
- Exam: unresponsive, tachypnea, regular tachycardia, otherwise unremarkable.
- **Laboratory Evaluation:**
- Serum: Na^+^ 142, K^+^ 5.9, Cl^-^ 116, HCO_3_^-^ 7, BUN 110, Creatinine 10.5 mg/dl, Glucose 101
- ABG: pH 7.05, pCO_2_ 27 mmHg

Solve the Acid-Base Disorder: ________________________________________________________

Write your management orders:

________________________________________________________________________________________________________________________________________________________________________________________________________________________________________________________________________________________________________________________________________

Likely Mechanism for the Disorder: _____________________________________________________

**Case 2**

- 58 year old male with acute abdominal pain and shock.
- Medical History: Tobacco use, hypertension, coronary artery disease
- In the ED found to have a ruptured aortic abdominal aneurysm.
- **Physical Examination:**
- VS: SBP 60 mmHg /palp; HR 140/min; RR 28/min; Temp 37.3 C; O2 Sat 98% on 10L
- Exam: unresponsive, tachypnea, regular tachycardia, pallor, cool extremities, mottling of the skin below the waist.
- **Laboratory Evaluation:**
- Serum: Na^+^ 138 mEq/L, K^+^ 5.9 mEq/L, Cl^-^ 100 mEq/L, HCO_3_^-^ 3 mEq/L, BUN 67 mg/dl, Cr 2.5 mg/dl
- ABG: pH 7.07, pCO_2_ 18 mmHg

Solve the Acid-Base Disorder: ________________________________________________________

Write your management orders:

________________________________________________________________________________________________________________________________________________________________________________________________________________________________________________________________________________________________________________________________________

Likely Mechanism for the Disorder: _____________________________________________________

**Case 3**

- 32 year-old male with poorly controlled HIV and a low CD4 count.
- Presents with 10 days of profuse, watery diarrhea
- Medical History: HIV, Hypertension
- **Physical Examination:**
- VS: BP 100/55 mmHg; HR 125/min; RR 28/min; Temp 37.3 C; O2 Sat 98% on Room air
- Exam: Well appearing, chest clear, regular tachycardia, no peripheral edema
- **Laboratory Evaluation:**
- Serum: Na^+^ 149, K^+^ 3.0, Cl^-^ 128, HCO_3_^-^ 12, BUN 13 mg/dl, Cr 0.8 mg/dl
- ABG: pH 7.30, pCO_2_ 25 mmHg
- Urine Electrolytes: Sodium 58, Potassium 11, Chloride 15

Solve the Acid-Base Disorder: ________________________________________________________

Write your management orders:

________________________________________________________________________________________________________________________________________________________________________________________________________________________________________________________________________________________________________________________________________

Likely Mechanism for the Disorder: _____________________________________________________

**Case 4**

- 22 year-old man goes to the beach with his friends.
- Later he has severe sunburn on most of his body.
- His mother applies a salve to the affected areas.
- The next day he feels unwell, weak, and short of breath.
- **Physical Examination:**
- VS: BP 125/65 mmHg; HR 75/min; RR 26/min; Temp 37.3 C; O2 Sat 98% on Room air
- Exam: Well appearing, tachypnea, sunburn noted on face, arms, and legs.
- **Laboratory Evaluation:**
- Serum: Na^+^ 141, K^+^ 4.2, Cl^-^ 104, HCO_3_^-^ 10, BUN 25 mg/dl, Cr 1.3 mg/dl
- ABG: pH 7.52, pCO_2_ 12 mmHg

Solve the Acid-Base Disorder: ________________________________________________________

Write your management orders:

________________________________________________________________________________________________________________________________________________________________________________________________________________________________________________________________________________________________________________________________________

Likely Mechanism for the Disorder: _____________________________________________________
