# Supplementary material for: The Treatment of Metabolic Acidosis: An Interactive Case-Based Learning Activity
Source: MedEdPORTAL. 2019 Sep 27;15:10835. doi: 10.15766/mep_2374-8265.10835 (PMC6897540; doi:10.15766/mep_2374-8265.10835)
Supplement: Supplementary file 1 — A. Approach to Acid-Base Disorders.mp4 B. Tale of Two Acidoses.mp4 C. IRAT Quiz.docx D. IRAT Quiz KEY.docx E. In-Class Cases.docx F. In-Class Cases Instructor Guide.docx [file mep-15-10835-s001.zip › F. In-Class Cases Instructor Guide.docx]

**The Treatment of Metabolic Acidosis: An Interactive Case-based Learning Activity.**

**Acid-Base Cases for Small Group Teaching**

**Case 1.**

- 61 year-old woman brought to ED by EMS after being found minimally responsive at home.
- Patient has not been seen by family for a few days. She has been taking NSAIDs for severe back pain.
- Medical History: chronic kidney disease, last serum creatinine 5.4 mg/dl
- EMS was called, patient was obtunded, tachycardic, tachypneic, taken to the ED.
- **Physical Examination:**
- VS: BP 100/62 mmHg; HR 110/min; RR 28/min; Temp 37.3 C; SpO2 98% on 3L NC
- Exam: unresponsive, tachypnea, regular tachycardia, otherwise unremarkable.
- **Laboratory Evaluation:**
- Serum: Na^+^ 142, K^+^ 5.9, Cl^-^ 116, HCO_3_^-^ 7, BUN 110, Creatinine 10.5 mg/dl, Glucose 101
- ABG: pH 7.05, pCO_2_ 27 mmHg

Solve the Acid-Base Disorder: Increased Anion Gap Metabolic Acidosis and Respiratory Acidosis

Write your management orders:

Order serum lactate, serum beta-hydroxybutyrate levels.

IV sodium bicarbonate: bolus 100mEq once followed by 150 mEq/L infused at 100ml/hour

Consult Nephrology for possible dialysis.

Likely Mechanism for the Disorder: Retention of non-volatile acids due to chronic renal failure and hypoventilation possibly secondary to uremic encephalopathy or respiratory muscle fatigue from compensation for severe metabolic acidosis. Correction of the metabolic acidosis with sodium bicarbonate therapy will raise the pH and reduce the respiratory demands for continued hyperventilation.

**Case 2**

- 58 year-old male with acute abdominal pain and shock.
- Medical History: Tobacco use, hypertension, coronary artery disease
- In the ED found to have a ruptured aortic abdominal aneurysm.
- **Physical Examination:**
- VS: SBP 60 mmHg /palp; HR 140/min; RR 28/min; Temp 37.3 C; O2 Sat 98% on 10L
- Exam: unresponsive, tachypnea, regular tachycardia, pallor, cool extremities, mottling of the skin below the waist.
- **Laboratory Evaluation:**
- Serum: Na^+^ 138 mEq/L, K^+^ 5.9 mEq/L, Cl^-^ 100 mEq/L, HCO_3_^-^ 3 mEq/L, BUN 67 mg/dl, Cr 2.5 mg/dl
- ABG: pH 7.07, pCO_2_ 18 mmHg

Solve the Acid-Base Disorder: Increased anion gap metabolic acidosis likely due to lactic acidosis and a respiratory acidosis likely due to reduced gas-exchange from poor perfusion plus respiratory muscle fatigue from compensation for a severe metabolic acidosis. The expected pCO_2_ is defined by Winter’s Formula: expected pCO_2_ = 1.5 x Bicarbonate + 8 ±2. In this case, expected pCO_2_ is defined by 1.5 x 3 + 8 = 12.5 ±2. Because the measured pCO2 is higher than the expected, there is a respiratory acidosis.

Write your management orders:

Order serum lactate and serum beta-hydroxybutyrate levels.

Place large bore IV access.

IV bolus 1000cc Normal Saline

Prepare 4 units packed red blood cells for transfusion

Emergency consult: Vascular Surgery

Likely Mechanism for the Disorder: Rapid production of new acid

**Case 3**

- 32 year-old male with poorly controlled HIV and a low CD4 count.
- Presents with 10 days of profuse, watery diarrhea
- Medical History: HIV, Hypertension
- **Physical Examination:**
- VS: BP 100/55 mmHg; HR 125/min; RR 28/min; Temp 37.3 C; O2 Sat 98% on Room air
- Exam: Well appearing, chest clear, regular tachycardia, no peripheral edema
- **Laboratory Evaluation:**
- Serum: Na^+^ 149, K^+^ 3.0, Cl^-^ 128, HCO_3_^-^ 12, BUN 13 mg/dl, Cr 0.8 mg/dl
- ABG: pH 7.30, pCO_2_ 25 mmHg
- Urine Electrolytes: Sodium 58, Potassium 11, Chloride 15

Solve the Acid-Base Disorder: Normal anion gap metabolic acidosis due to diarrhea (bicarbonate losses). There is a normal (negative) urine anion gap.

Write your management orders:

IV sodium bicarbonate: bolus 100mEq (two ampules) followed by 150 mEq/L infused at 100ml/hour

Likely Mechanism for the Disorder: loss of bicarbonate rich fluid (diarrhea/GI losses)

**Case 4**

- 22 year-old man goes to the beach with his friends.
- Later he has severe sunburn on most of his body.
- His mother applies a salve to the affected areas.
- The next day he feels unwell, weak, and short of breath.
- **Physical Examination:**
- VS: BP 125/65 mmHg; HR 75/min; RR 26/min; Temp 37.3 C; O2 Sat 98% on Room air
- Exam: Well appearing, tachypnea, sunburn noted on face, arms, and legs.
- **Laboratory Evaluation:**
- Serum: Na^+^ 141, K^+^ 4.2, Cl^-^ 104, HCO_3_^-^ 10, BUN 25 mg/dl, Cr 1.3 mg/dl
- ABG: pH 7.52, pCO_2_ 12 mmHg

Solve the Acid-Base Disorder: Increased Anion Gap metabolic acidosis and respiratory Alkalosis, due to salicylate toxicity

Write your management orders:

IV sodium bicarbonate 150 mEq/L infused at 100ml/hour

Order serum lactate, serum beta-hydroxybutyrate levels.

Check serum salicylate concentration.

Consult Nephrology for possible dialysis.

Likely Mechanism for the Disorder: Ingestion/production of new acid
